# Reinvestigation of the origins of pineal meningiomas based on its related veins and arachnoid membranes

**DOI:** 10.1186/s12883-020-01783-4

**Published:** 2020-05-20

**Authors:** Lei Yu, Berdimyrat Orazmyradov, Songtao Qi, Ye Song, Luxiong Fang

**Affiliations:** Department of Neurosurgery, Nanfang Hospital, Southern Medical University, 1838 Guangzhou Dadao Bei Street, Guangzhou, 510515 P. R. China

**Keywords:** Meningiomas, Pineal region, Velum interpositum, Falcotentorial, POPPEN approach, Arachnoid membranes

## Abstract

**Background:**

A series of patients harboring pineal region meningiomas were respectively analyzed to explore the origin of these tumors and the true meaning of the term “velum interpositum (VI) meningiomas”.

**Methods:**

21 patients with pineal meningiomas underwent operation in Nanfang Hospital of Southern Medical University from January 2005 to December 2016 were retrospectively included to analyze the clinical features, imaging findings and surgical video data of these patients. According to the method of literature, the data of this group were also divided into falcotentorial (FT) meningiomas and VI meningiomas, and the differences between the two types of tumors were compared.

**Results:**

Among the 21 cases of tumor, there were 12 cases of FT meningiomas, including 4 cases originating from cerebral falx, 4 cases from tentorium of cerebellum and 4 cases from straight sinus; there were 9 cases of VI meningiomas, 7 of which originated from the arachnoid sleeve of the Galen vein, 1 from the posterior part of the internal cerebral vein and 1 from the posterior surface of the pineal gland. Postoperative pathological examination showed meningiomas in all the 21 patients, including 16 cases of total resection and 5 cases of subtotal resection. Postoperatively limitation of binocular vertical motion was found in 3 cases, homotropic hemianopia in 7 cases, hemiplegia in 1 case and death in 1 case.

**Conclusions:**

This study suggests that pineal meningiomas are more suitable to be described by FT meningioma and meningiomas of the arachnoid of the pineal region by analyzing the origin of tumors. The term “VI meningiomas” can only reflect a part of meningiomas of the arachnoid of the pineal region. Before the removal of pineal meningiomas, more attention should be paid to the effects of the two types of tumors on the Galen vein and the straight sinus, and the establishment of venous collateral circulation.

## Background

Pineal region tumors are located deeply and adjacent to important anatomical structures. Surgical treatment requires a clear understanding of the origin of tumors [1–3]. There are currently different classifications and nomenclatures for pineal region meningiomas, which can be divided into falcotentorial (FT) meningiomas and primary pineal region meningiomas, or into FT and velum interpositum (VI) meningiomas [4, 5]. However, the clinical classification of pineal region meningiomas is limited by the low incidence [2], and the current classification method need be further discussed. Therefore, it is of great significance to re-investigate the origin and classification of pineal region meningiomas. The relationship between tumors and internal cerebral veins (ICVs) and Galen vein (GV), and between tumors and tentorium of cerebellum and cerebral falx were emphatically analyzed by studying a group of cases. The study is to explore the origin of pineal region meningiomas and the true meaning of the term “VI meningiomas”.

## Methods

### Patient population

From January 2005 to December 2016, we retrospectively included 21 patients with pineal meningiomas treated by surgery in Nanfang Hospital of Southern Medical University. The clinical records, neuroimaging studies, and follow–up data of the treated patients were reviewed. There were 14 women and 7 men whose ages ranged from 20 to 67 years (mean 48.6 years). The clinical manifestations included headache and dizziness in 16 cases, unstable gait in 3 cases, blurred vision in 1 case, and tumors occasionally observed in 1 case due to head trauma.

### Surgical approach

All the 21 patients underwent microsurgical removal of the tumor. The occipital-transtentorial approach (Poppen approach, ¾ prone position) was used in all patients, and we preferred this approach for pineal region tumors. Cerebrospinal fluid was fully released to facilitate retraction of the occipital lobe. The dura was incised in a + −shaped fashion to the angle between the superior sagittal sinus and transverse sinus. After the occipital pole was retracted gently toward the parietal lobe, the tentorium was incised 1–1.5 cm paramedian and parallel to the straight sinus to fully expose the tumor. For patients with supratentorial and parafalx tumors, transtentorial or combined transfalx approach was used to resect and locate the origin of the tumors. For patients with no clear tumor growth on the supratentorial surface, the tentorium of cerebellum was incised beside the straight sinus to resect the subtentorial tumors, and the tumors were gradually pulled toward the center to determine the origin of the tumors. For these infratentorial tumors, the infratentorial supracerebellar approach is indeed more appropriate, but due to the limitations of our conditions and experiences, the approach is less used in our department. The occipital-transtentorial approach has a wide range of indications and is fully competent for the removal of these pineal region tumors. This was confirmed by our group of cases and our published literature [3].

## Results

### Neuroimaging resullts

All patients were evaluated using magnetic resonance imaging (MRI) and computed tomography (CT) scan. For MRI all patients underwent T1-and T2-weighted imaging, T1-contrast-enhanced sequences and magnetic resonance venogram (MRV). Most of the tumors were round or oval, and 2 were lobulated with a maximum diameter of 2.2–5.8 cm. On CT images, tumors show clear boundaries and uniform density. 9 cases showed equal density, 8 cases showed slightly high density, and 4 cases showed slightly low density. There were no necrotic cysts in the tumors, but 3 cases had small calcification. In 5 cases, single calcification foci (pineal calcification) were found around the tumors, mostly located in the anterior superior or anterior lateral. MRI showed that 17 cases of tumors were located in the midline with bilateral symmetry, and 4 cases were inclined to one side. Homogeneous enhancement was showed in 15 cases, enhancement was more obvious in periphery than in the central part in 2 cases, and heterogeneous enhancement was found in 4 cases. In one case the tumors were multiple, located in the pineal region and the right anterior clinoid process; the remaining 20 tumors were located in the pineal region, and no intracranial or spinal cord metastasis was found. Preoperative imaging diagnosis included meningioma in 14 cases, pineal parenchymal tumors in 6 cases and germ cell tumors in 1 case.

### The relationship between tumors and GV and the dura of FT junction

**Fig. 1:**
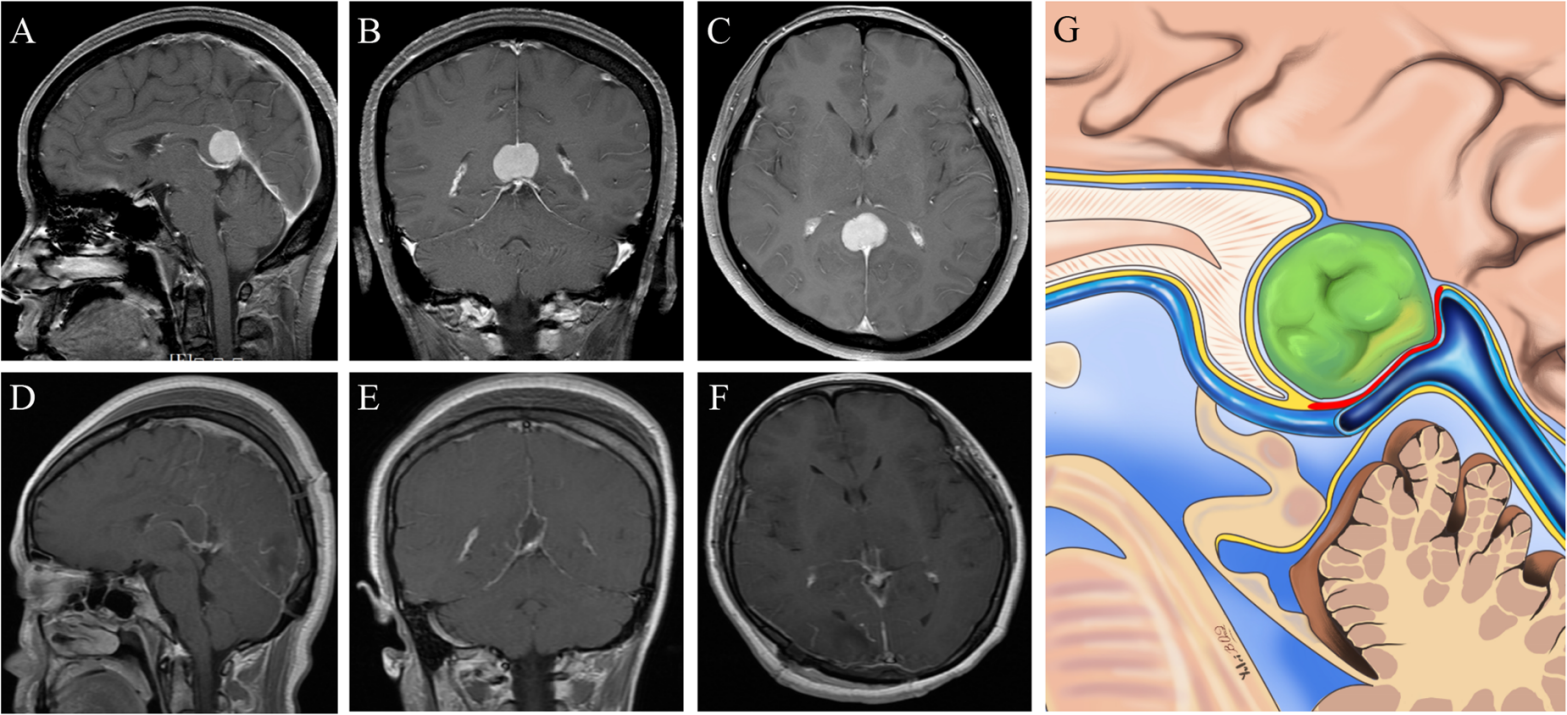
Radiological imaging of the tumor in the anterior superior part of the ICVs and the GV. **a-c:** Preoperative images showing a pineal region meningioma. **d-f:** Postoperative images showing gross-total removal of the tumor. **g:** Schematic representation of the microanatomical relationship between the tumor origin and arachnoid membranes. (Yellow lines represent the arachnoid membrane; Red line represent possible tumor attachment)

**Fig. 2:**
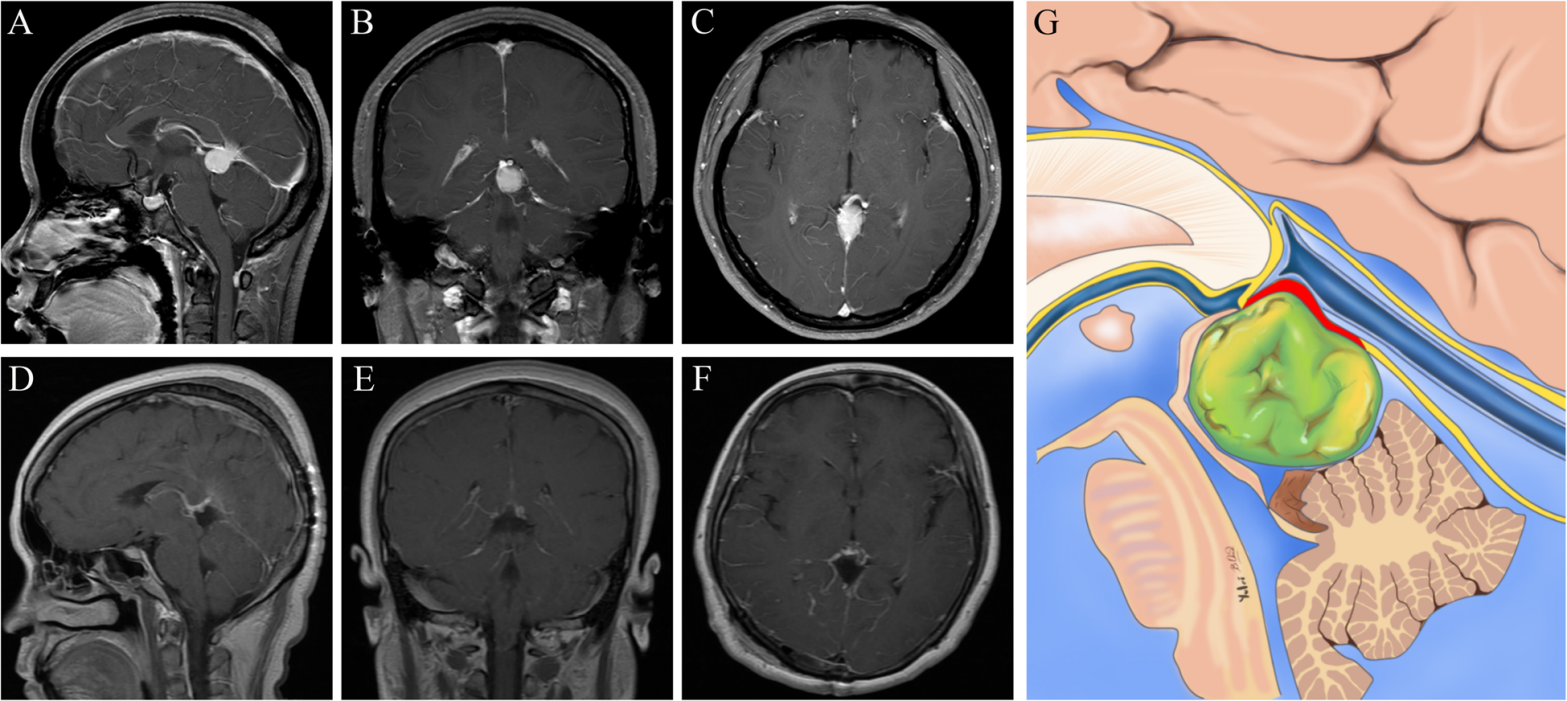
Radiological imaging of the tumor in the posterior inferior part of the ICVs and the GV. **a-c:** Preoperative images showing a pineal region meningioma. **d-f:** Postoperative images showing gross-total removal of the tumor. **g:** Schematic representation of the microanatomical relationship between the tumor origin and arachnoid membranes. (Yellow lines represent the arachnoid membrane; Red line represent possible tumor attachment)

**Fig. 3:**
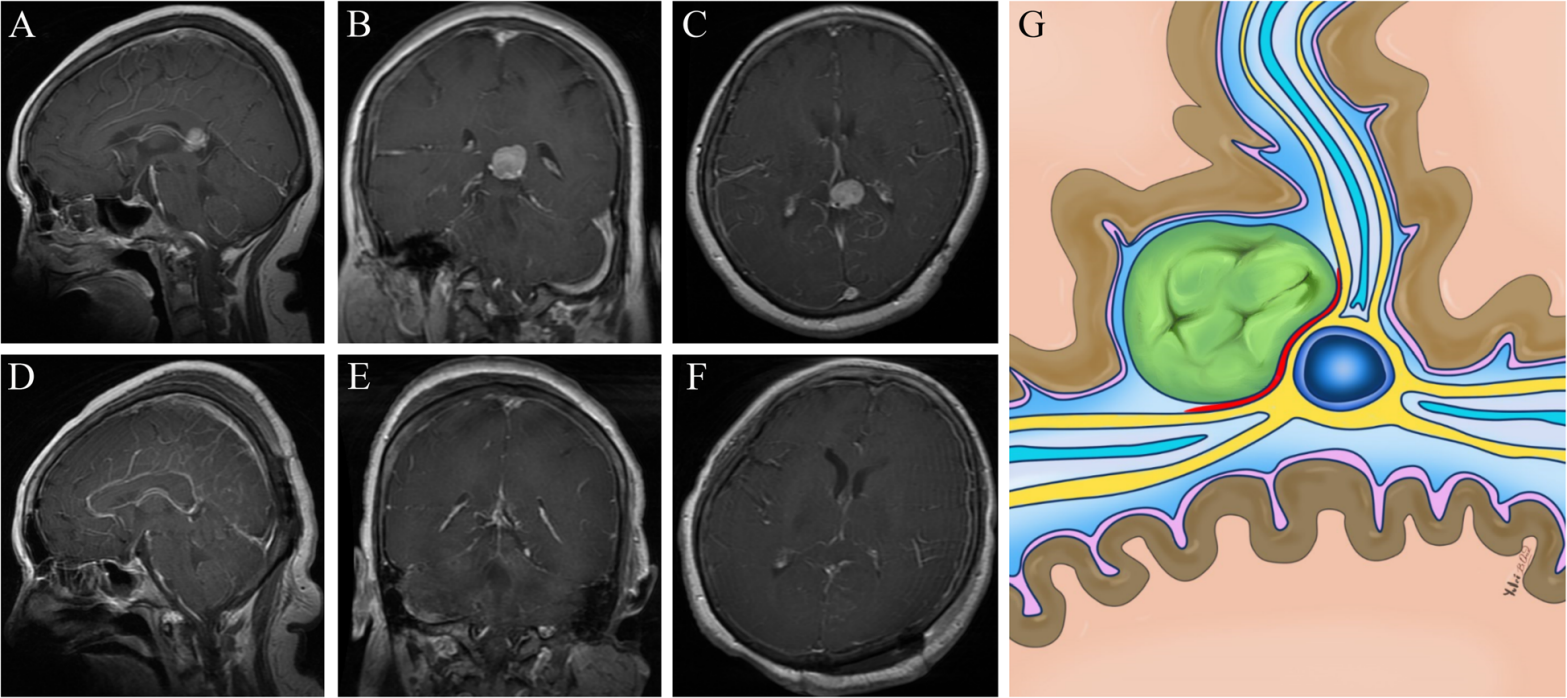
Radiological imaging of the tumor on the lateral side of the ICVs and the GV. **a-c:** Preoperative images showing a pineal region meningioma **d-f:** Postoperative images showing gross-total removal of the tumor. **g:** Schematic representation of the microanatomical relationship between the tumor origin and arachnoid membranes. (Yellow lines represent the arachnoid membrane; Red line represent possible tumor attachment)

In the 21 patients, the relationships between tumors and GV (Figs. 1, 2, 3), tentorium of cerebellum, and cerebral falx were summarized in the Table 1. MRV showed that the GV and the straight sinus were not visualized in 12 cases, stenosis of the GV in 3 cases, and displacement of the GV without obvious abnormality in 3 cases. Of the 12 patients whose GV could not be visualized, 5 had dilated veins on the medial parietal lobe, which was considered as venous collateral circulation. Combined with CT examination, 8 cases showed displaced pineal gland located in the anterior part of the tumor. 15 cases had hydrocephalus and ventricular enlargement mildly to moderately.

**Table 1:** The relationship between tumors and GV, tentorium of cerebellum, and cerebral falx

	No. of Cases
Tumors and GV	
Posteriorly and inferiorly to GV	12
Anteriorly and superiorly to GV	5
Laterally to GV	3
GV completely wrapped	1
Contact area between tumors and adjacent structures	
Contact area between tumor and tentorium	4
Contact area between tumor and FT junction	5
Contact area between tumor and inferior surface of straight sinus	5
Contact area between tumor and GV	4
Contact area between tumor and posterior segment of GV and ICVs	2
Contact area between tumor and posterior segment of ICVs and pineal gland	1
Enhancement of FT junction	
Yes	12
No	9
Dural tail sign in FT junction	
Yes	5
No	16

### The origin of tumor

To understand the origin of the tumors, the authors reviewed the surgical records and video recordings of the 21 patients. (1) Tumors were found supratentorially in 9 cases: 3 cases originated from tentorium and grew under the tentorium or through tentorium hiatus to supratentorial area on the supratentorial surface of tentorium and on the lateral side of cerebral falx; After retracting the medial occipital lobe, tumors were seen beside cerebral falx just in front of tentorium cerebelli hiatus in 6 cases and were resected by incising cerebral falx. Four tumors originated from cerebral falx and two from the arachnoid sleeve of the GV. (2) 12 cases of tumors were located entirely under the tentorium by incising the tentorium near the straight sinus. Among them, tumors originated beside the straight sinus in 1 case and from the surface of the inferior straight sinus in 4 cases. The other 7 cases of tumor had no direct contact with tentorium. Five of them originated from the arachnoid sleeve of the GV, one from the posterior segment of the ICV and one from the arachnoid membrane on the surface of the pineal gland irrelevant of the GV and the ICV. The above results were summarized in the Table 2. In general among the 21 cases of tumor, there were 12 cases of FT meningiomas, including 4 cases originating from cerebral falx, 4 cases from tentorium of cerebellum and 4 cases from straight sinus; there were 9 cases of VI meningiomas, 7 of which originated from the arachnoid sleeve of the GV, 1 from the posterior part of the ICV and 1 from the posterior surface of the pineal gland.

**Table 2:** The origin of tumor

	No. of Cases
Supratentorially	9
tentorium	3
cerebral falx	4
arachnoid sleeve of the GV	2
Infratentorially	12
tentorium	4
inferior surface of SS	1
arachnoid sleeve of GV	5
posterior segment of ICV	1
arachnoid membrane on the surface of pineal gland	1

### Operative results and outcome

By reviewing the pathological section postoperative pathological examination showed meningiomas in all the 21 patients (meningothelial 9 cases, fibrous 5, transitional 3, fsammomatousa 1, angiomatous 1, and anaplastic 2, according to the 2016 World Health Organization Classification of Tumors of the Central Nervous System), including 16 cases of total resection and 5 cases of subtotal resection. Postoperative death occurred in 1 case with giant FT meningiomas, which was caused by brain swelling resulting from venous collateral circulation destruction. One patient also with giant FT meningiomas was paralysed after operation and returned to normal 3 weeks later. Homotropic hemianopia occurred in 7 patients after operation. Five of them returned to normal after 1 month, one improved and one did not take a favourable turn. There were 3 cases of bilateral vertical motion limitation of eyes after operation with no improvement during the follow-up period. Before discharge, the symptoms of headache and dizziness were alleviated, and the walking function was improved in all the patients. Postoperative imaging examination showed that hydrocephalus was all successfully arrested. The patients with subtotal resection of tumors were followed by gamma knife treatment. 16 patients were followed up for 6 months to 9.5 years, with an average of 51.0(±8.7) months. Tumor recurrence occurred in 2 cases, all of which were FT meningiomas and the tumors were stable after gamma knife treatment.

## Discussion

Meningiomas in pineal region are rare, accounting for 0.3–1.0% of intracranial meningiomas and 2–8% of pineal region tumors [6, 7]. At present, the classification of pineal meningiomas into FT meningiomas and VI meningiomas is generally accepted. FT meningiomas originate from the arachnoid membrane attached to the FT junction and protrude into the pineal region. Therefore the tumors are directly related to the the dura of FT junction. However the VI meningioma originated from the arachnoid membrane covering the VI, located in the pineal region, and had no direct ralationship with the the dura of FT junction. Therefore, the main difference between FT meningiomas and VI meningiomas is whether the tumors are directly related to the dura of FT junction [4, 8]. Unfortunately, it is still difficult to distinguish between FT meningiomas and VI meningiomas even by modern imaging methods, so further confirmation is needed during surgical operation.

The VI meningiomas are rare clinically and only scattered case reports were found in literature review. According to the data provided by Champagne and Bojanowski, up to 2014, there were only 22 cases reported worldwide over a 70-year period [8]. In 2014, Nowak et al. reported 6 cases of pineal meningiomas treated surgically during the last 20 years, of which 2 cases were VI meningiomas, accounting for 1/3 [4]. It can be seen that the proportion of VI meningiomas in pineal meningiomas is not low. These contradictory data indicate that the incidence of the VI meningioma is underestimated or overestimated. The author believes that the reason is that the accurate concept of the VI meningioma is still not clear enough.

**Fig. 4:**
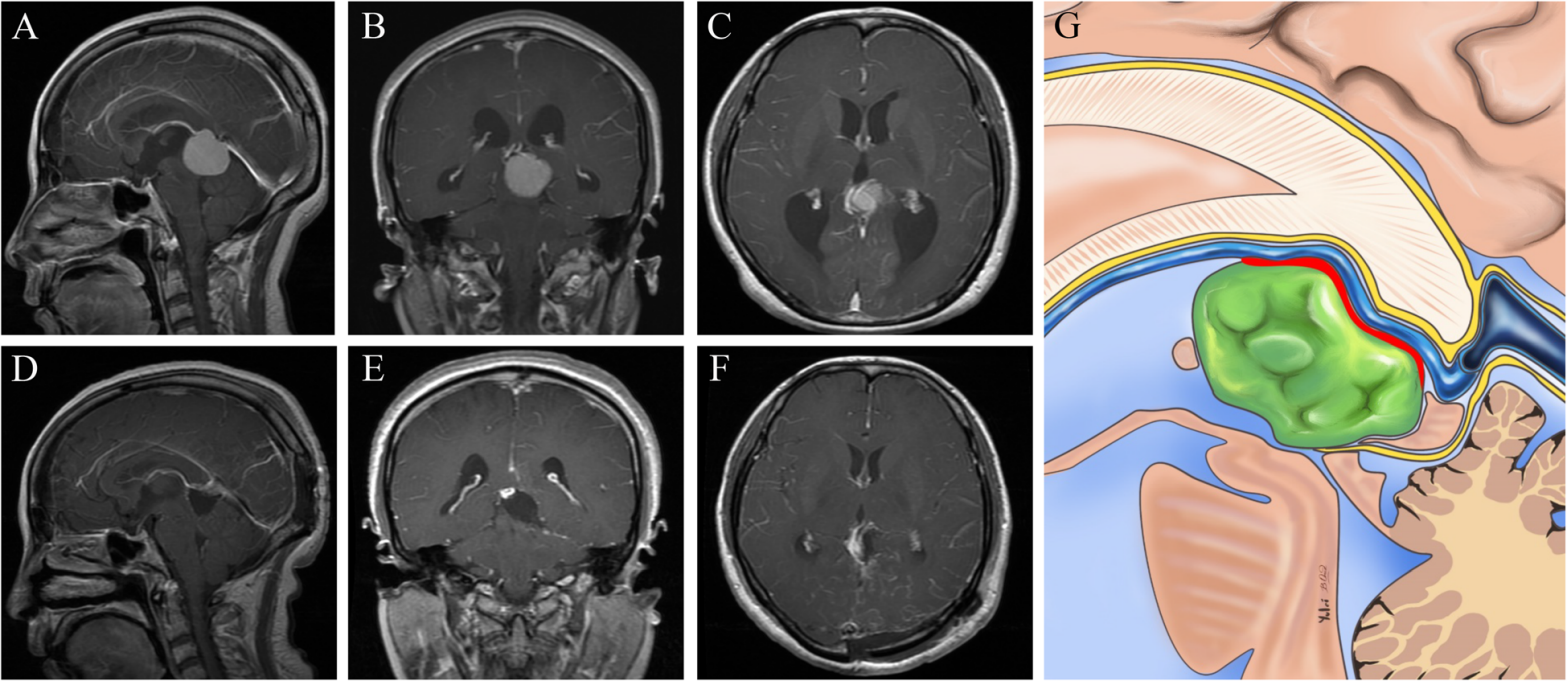
Radiological imaging of the tumor deriving from the arachnoid architecture within the velum interpositum. **a-c:** Preoperative images showing a pineal region meningioma. **d-f:** Postoperative images showing gross-total removal of the tumor. **g:** Schematic representation of the microanatomical relationship between the tumor origin and arachnoid membranes. (Yellow lines represent the arachnoid membrane; Red line represent possible tumor attachment)

The origin of meningiomas in pineal region was studied by imaging examination combined with intraoperative verification among this group of patients. Of the 21 cases of tumor, 12 cases were confirmed to be FT meningiomas, which was directly related to the the dura of FT junction; 9 cases should be classified as VI meningiomas, which had no direct relationship with the dura of FT junction according to the current commonly used classification of pineal meningiomas. However, only one case of the 9 patients originated from the posterior segment of the ICVs within the VI **(**Fig. 4), the other case from the posterior part of the pineal gland, and the other 7 cases from the arachnoid sleeve of the GV.

Since meningiomas originate from the cap cells of inner or outer arachnoid, those in the pineal region can originate from two sites [9]. First, they can arise at the FT junction from the arachnoid layer, which tightly follows the dura. Although FT meningiomas are the most common, they are not considered to be “true” pineal region meningiomas because they do not originate from the region itself and just grow toward it [10, 11]. Second, they can derive from the arachnoid envelope over the pineal region (AEPG) and the arachnoid architecture within the VI [9, 12]. Therefore it is not appropriate to divide pineal meningiomas into FT meningiomas and VI meningiomas because the term “VI meningiomas” reflects only a part of meningiomas of the arachnoid of the pineal region. Although it is worth discussing, the term “ VI meningiomas “ is still used in this paper in order to be consistent with the literature. In fact, the term “VI meningiomas” appeared before the advent of CT and MRI the definition of VI meningiomas in the previous literature was vague. Lozier, A. P stated that tumors that arised from the ventral tela choroidea, the dorsal tela choroidea, or the posterior tenia fornicis (the site of attachment of the dorsal tela choroidea) in the third ventricle might be referred to as VI meningiomas [6]. Nowak, A., et al. suggested that VI meningiomas, without dural attachment in the pineal region, arose from the posterior portion of the velum interpositum [4]. Bojanowski found that VI meningiomas were more commonly found on the inferior leaflet of the VI because most VI meningiomas arose not from the VI itself, but from the cap cells presented in the choroid plexus, which was adjacent to the inferior leaflet of the VI [8]. The reason why the definition of VI meningiomas was vague was that there existed controversy on the arachnoid architecture within the velum interpositum. In fact, there are two arachnoid layers within the VI. The dorsal layer of arachnoid membrane envelops the ICVs while the ventral layer of arachnoid membrane is a direct anterior extension of the APEG and covers the midline inferior layer of tela choroidea [12]. So the meningiomas that really originate from these two parts of arachnoid cap cells can be called VI meningiomas. Meningiomas that actually originate from these two sites are very rare clinically. Most of the so-called “VI meningiomas” actually originate from the arachnoid sleeve of the GV (the posterior part of APEG). As in our case group, only one case of the 9 patients, which had no direct relationship with dura of the FT junction, originated from the dorsal layer of arachnoid membrane within the VI, and the other 8 cases from the posterior part of APEG.

Efforts to differentiate FT meningiomas from VI meningiomas are of surgical significance. The arachnoid interface of FT meningiomas was clear, while most of the interface of VI meningiomas were damaged. In addition to their different relationship with the dura of FT junction, there are also fundamental differences in the blood supply of tumors. The blood supply of FT meningiomas may derive from the meningohypophyseal trunk, the meningeal branch of the external carotid artery, the small branch of the posterior cerebral artery, and the branches of the posterior medial and lateral choroidal arteries [2]. The above arteries participate in blood supply alone or together and the blood supply of tumor is abundant. Cutting off the the dura of FT junction before tumor resection can reduce bleeding during tumor resection [13]. The VI meningiomas is usually supplied only by the posterior choroidal artery, and the blood supply is generally not rich. However, if the supratentorial approach is used, the contralateral feeding artery is not easily blocked in the early stage of operation.

**Fig. 5:**
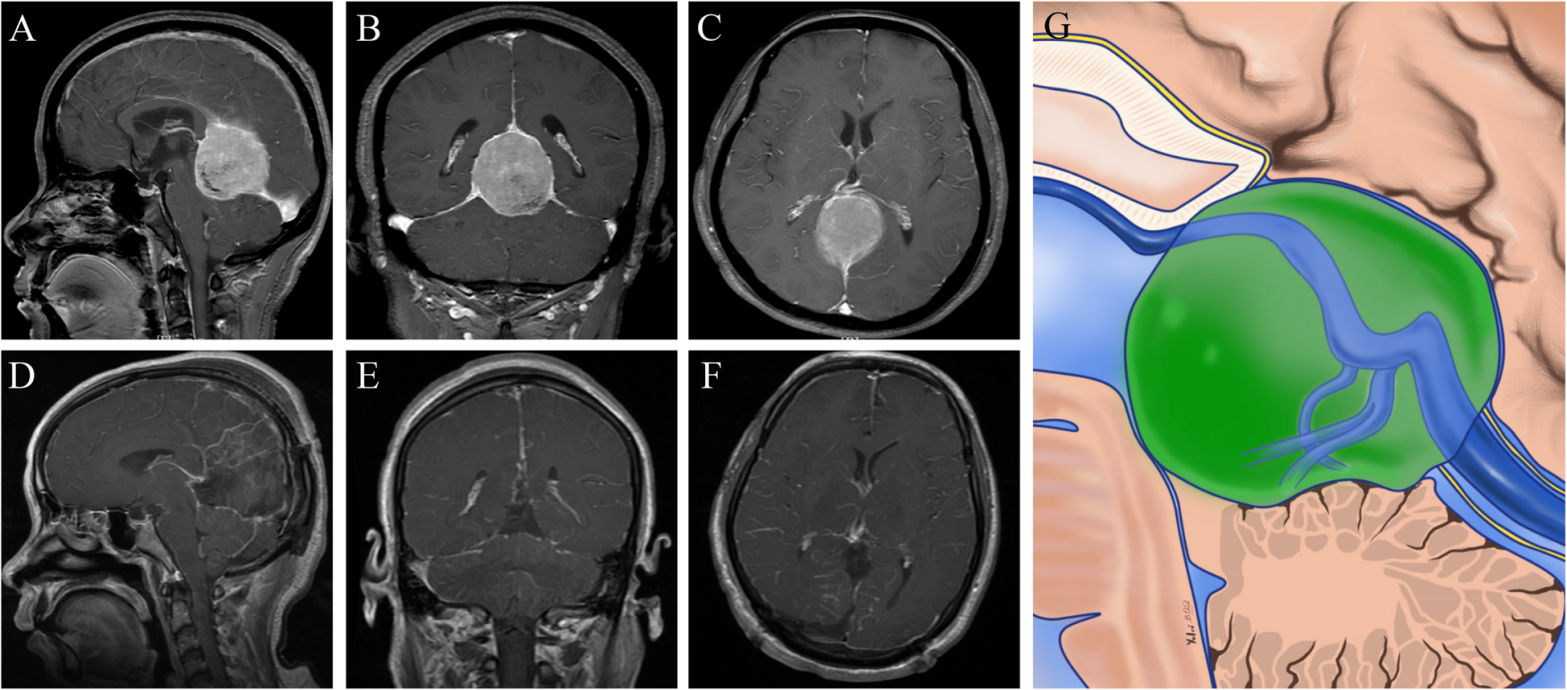
Radiological imaging of the tumor completely encapsulating the deep venous system. **a-c:** Preoperative images showing a pineal region meningioma **d-f:** Postoperative images showing gross-total removal of the tumor. **g:** Schematic representation of the microanatomical relationship between the tumor origin and arachnoid membranes. (Yellow lines represent the arachnoid membrane)

Before the removal of pineal meningiomas, more attention should be paid to the effects of tumors on the GV and the straight sinus, and the establishment of venous collateral circulation. It had been reported that there were two spatial relationships between tumors and veins, either in the anterior superior part of the ICVs and the GV, or in the posterior inferior part of these veins [10]. However, Blasco proposed dividing FT meningiomas into 4 subtypes according to the Bassiouni classification and its relationship with the deep venous system: (A) FTM type I with inferior venous displacement, (B) FTM type II with superior venous displacement, (C) FTM type III with contralateral venous displacement, and (D) FTM type IV with growth over the straight sinus and superolateral venous displacement [7, 14]. In addition to the above spatial relationships the veins could be encapsulated totally by the tumor in the present group of patients **(**Fig. 5**)**. The veins should be kept away in the selection of surgical approaches.

Compared with other pineal region tumors, meningiomas seem to have more obvious effects on veins and venous sinuses. The proportion of GV and straight sinuses that cannot be visualized on MRV is higher, which indicates that most meningiomas affect venous reflux. Intraoperative observation showed that the two types of meningiomas had different effects on veins. FT meningioma could invade venous sinuses and GV, and even wrap the GV completely in the tumor. If the veins were still functioning, it might be a wise choice to protect the veins from being electrocoagulated and perform a subtotal resection of the tumor with residue. The VI meningiomas only compress and displace the veins, and do not invade the veins. After the removal of tumor, the veins can be reopened. Therefore, even if the veins are not visualized before operation, the VI meningiomas should be treated according to the patency of the vein before operation, and it should not be cut off rashly unless the veins are completely occluded. The collateral circulation may be established to compensate for the obstruction of the straight sinus and the GV. The present study found that FT meningioma showed signs of venous vasodilation on the medial surface of the anterior occipital lobe and the posterior parietal lobe. However, similar imaging findings were not found in VI meningiomas, suggesting that there were differences in venous collateral circulation between the two types of meningiomas, suggesting that “VI meningiomas” had less influence on venous reflux. The POPPEN approach is more likely to destroy venous collateral circulation than subtentorial approach and therefore great attention should be paid to the protection of medial occipital vein and tentorial sinus during operation [1, 3, 13, 15]. In principle, the protection of the GV and collateral circulation is particularly important and if there are residual tumors, gamma knife can be used for the follow-up treatment.

## Conclusion

In conclusion, this study suggests that pineal meningiomas are more suitable to be described by FT meningiomas and meningiomas of the arachnoid of the pineal region by analyzing the origin of tumors. The term “VI meningiomas” can only reflect a part of meningiomas of the arachnoid of the pineal region. Before the removal of pineal meningiomas, more attention should be paid to the effects of the two types of tumors on the Galen vein and the straight sinus, and the establishment of venous collateral circulation.

## Data Availability

The datasets are available from the corresponding author on reasonable request.

## Abbreviations

VI: Velum interpositum
FT: Falcotentorial
ICVs: Internal cerebral veins
GV: Galen vein
CT: Computed tomography
MRV: Magnetic resonance venogram
AEPG: Arachnoid envelope over the pineal region
